# Factors associated with the failure of first and second-line antiretroviral therapies therapy, a case control study in Cambodian HIV-1 infected children

**DOI:** 10.1186/s13104-016-1884-y

**Published:** 2016-02-05

**Authors:** Hubert Barennes, Kang Virak, François Rouet, Yves Buisson, Michel Strobel, Ung Vibol

**Affiliations:** Agence Nationale de Recherche sur le VIH et les Hépatites, Preah Monyvong Blvd, Phnom Penh, Cambodia; Institut de la Francophonie pour la Médecine Tropicale, Vientiane, Lao People’s Democratic Republic; ISPED, Centre INSERM U897-Epidemiologie-Biostatistique, Univ. Bordeaux, 33000 Bordeaux, France; Epidemiology Unit, Pasteur Institute, Phnom Penh, Cambodia; Virological Unit, Pasteur Institute, Phnom Penh, Cambodia; University of Health Science, Phnom Penh, Cambodia

**Keywords:** AIDS, Antiretroviral therapy, Cambodia, Children, CD4, HIV, Low income countries, NCHADS, Orphans, Virological failure

## Abstract

**Background:**

Little is known about the efficacy of first and and second-line antiretroviral therapies (ART) for HIV-1 infected children in resource limited Southeast Asian settings. Previous studies have shown that orphans are at a higher risk for virological failure (VF) in Cambodia. Consequently most of them required transfer to second-line ART. We assessed the factors associated with VF among HIV-1 infected children who were either under first-line (mostly 3TC + D4T + NVP) or under second-line (mostly ABC + DDI + LPV) therapies at a referral hospital in Cambodia.

**Methods:**

A case-control study was conducted from February to July 2013 at the National Pediatric Hospital among HIV-1 infected children (aged 1–15 years) under second-line ART (cases) or first-line (matched controls at a ratio of 1:3) regimens. Children were included if a HIV-1 RNA plasma viral load (VL) result was available for the preceding 12 months. A standardized questionnaire explored family sociodemographics, HIV history, and adherence to ART. Associations between VF (HIV-1 RNA levels ≥1000 copies/ml) and the children’s characteristics were assessed using bivariate and multivariate analyses.

**Results:**

A total of 232 children, 175 (75.4 %) under first-line and 57 (24.6 %) under second-line ART, for a median of 72.0 (IQR: 68.0–76.0) months, were enrolled. Of them, 94 (40.5 %) were double orphans and 51 (22.0 %) single orphans, and 77 (33.2 %) were living in orphanages. A total of 222 children (95.6 %) were deemed adherent to ART. Overall, 18 (7.7 %; 95 % CI 4.6–11.9) showed a VF, 14 (8.6 %; 95 % CI 4.8–14.0) under first-line and 4 (7.0 %; 95 % CI 1.9–17.0) under second-line ART (p = 0.5). Their median CD4 percentage was 8 % (IQR 2.9–12.9) at ART initiation. Children under second-line ART were older; more often double orphans, and had lower CD4 cell counts at the last control.

In the multivariate analysis, having the last CD4 percentage below 15 % was the only factor associated with VF for ART regimen separately or when combined (OR 40.4; 95 % CI 11–134).

**Conclusions:**

The pattern of risk factors for VF in children is changing in Cambodia. Improved adherence evaluation and intensified monitoring of children with low CD4 counts is needed to decrease the risk of VF.

**Electronic supplementary material:**

The online version of this article (doi:10.1186/s13104-016-1884-y) contains supplementary material, which is available to authorized users.

## Background

By the end of 2013, of the 35.3 million people living with HIV in the world, 3.3 million were children. Improved access to services for prevention of mother-to-child transmission of HIV and improved availability of antiretroviral therapy (ART) have reduced the number of new HIV infections by 50 % and AIDS-related deaths by 20 % among children [1].

The number of children, less than 15 years, receiving ART in low-and middle-income countries increased from 566,000 in 2011 to 630,000 in 2012 but this increase remains far below that of adult patients [2, 3]. An estimated 200,000 children with HIV live in Southeast Asia. Of them, 46,000 (23 %) receive ART, 21,000 were newly infected and 13,000 died in 2012 [4]. Good ART outcomes have been reported from pediatric HIV/AIDS programs in low resource settings, comparable to those in high-income countries [5].

Children on ART pose crucial concerns in low resources settings [4, 5]. First, they require long-term therapy with unknown long-term side effects. Second, the scarcity of pediatric formulations and inadequate dosage guidelines for specific antiretrovirals (ARVs) and age groups may contribute to suboptimal plasma drug levels. Third, these children, especially teenagers, have decreased adherence to treatment. All these factors can result in rapid HIV drug resistance (HIV DR).

Children are twice as likely to experience virological failure (VF) to ART as compared with adults after 5 years of treatment and 13–53 % of them are expected to experience VF within the first year [6]. Therefore, they are at a higher risk of developing HIV DR, particularly if failure is diagnosed late [7]. HIV DR genotyping has become a standard of care in HIV infection management in developed countries [6] but its availability remains infrequent in the developing world [8].

Over the last decade, Cambodia’s human immunodeficiency virus (HIV) program (NCHADS, National Center for HIV/AIDS, Dermatology and STD, Sexually Transmitted Diseases) has been quite successful. The prevalence of HIV infection decreased from 2.4 % in 1998 to 0.7 % in 2012 [9, 10]. The number of children living with HIV was estimated at 8512 and 4052 (47.6 %) were under ART in 2013 [9, 11, 12].

Several studies have reported the high effectiveness of ART in Cambodia for durations ranging from 12 to 36 months [13–15]. Compared to ART children, pre-ART children have a 4:1 mortality ratio and a 13:8 ratio of loss to follow-up [14, 15]. Being an orphan was considered an important predictor of VF [13].

Data on pediatric treatment monitoring and resistance in first-line and second-line failures are limited in Cambodia. Based on the WHO 2010 pediatric guidelines, without routine viral load monitoring, treatment failure were misclassified for children on first-line therapy [16, 17]. In addition, extensive drug resistance to first-line ART was described among 51 HIV-infected children, who were undetected as first-line ART failures under the WHO 2010 guidelines [18]. Similarly the HIV/Hepatitis laboratory of the Pasteur Institute in Cambodia reported the occurrence of mutations in children from routine samples collected between December 2004 and January 2011 [17]. Since then, children were switched to second-line ART primarily based on clinical and immunological criteria, following national pediatric guidelines [19]. This is the first study to compare the current profile of children under first-line and second-line ART in Cambodia and to assess the evolution of factors associated with VF in a pediatric referral hospital.

## Methods

### Ethics statement

The study was authorized by the National Pediatric Hospital (NPH) authorities. Ethical approval was granted by the Cambodian and Lao Medical Ethics Committees because the study was conducted in Cambodia as part of a master study from the “Institut de la Francophonie pour la Médecine Tropicale” (IFMT, Vientiane, Laos). The study complied with the Cambodian law on personal protection of people living with HIV. Children and parents/guardians were informed about the study in Khmer language and given an information paper describing it. They were included if they consented to participate and if their parent/guardians gave informed consent in writing. Confidentiality was guaranteed and interviews were conducted by a clinical investigator in a private room. Attention was paid to not disclosing the reason for the visit, or the child’s status, during home visits. Data was recorded anonymously.

### Study site

The study was conducted from February to July 2013 in the HIV clinic of the NPH in Phnom Penh during children’s routine visits. The NPH was the first setting to start ART in 2004. At the time of the study NPH provided ART to 1300 HIV children, including 107 on second-line ART. Children were monitored every 3 months at the outpatient department for clinical status, adherence and counseling and CD4 cell counts.

Plasma HIV-1 viral load (VL) was measured according to the Cambodian National Guidelines for Management of Pediatric HIV recommendation once within the first 6 months of starting ART and then every 12 months. HIV-1 genotyping was not routinely available for children.

### Study procedures and questionnaires

Cambodian children between 12 months and 15 years attending the HIV clinic were eligible if they had been under second-line ART for at least 6 months and if VL had been performed after the start of second–line line and within the 12 months prior to the survey. For each child on second-line ART, 3 children on first-line ART and of a similar age ±1 years and with a VL conducted within the last 12 months were randomly selected among those attending the clinic the same day.

An interview was conducted with the parent/guardian and child in Khmer language. The questionnaire included sociodemographic items about the household, parental resources (if available), access to care and compliance with ART (Additional file 1).

For children younger than 7 years, the parent/guardians were asked questions. Children over 7 years old were asked these questions directly in the presence of their parent/guardian.

Data relating to the child’s HIV status, disease history and treatment was retrieved from the hospital records after the interview. Adherence to treatment was evaluated by the recall of missed medication intake during the previous 4 days, within the previous month and by counting the tablets at home. The tablet counting was performed after parental agreement, on appointment with parent/caregivers within 1 month after the first meeting. Adherence variables were dichotomized as complete (100 %) vs. incomplete (<100 %) [20].

### Definitions

Single orphans were children who had lost one parent and double orphans were children who had lost both.

Virological failure was defined as plasma HIV-1 RNA level ≥1000 copies/mL. For children on second-line therapy, the VL had to be done after the start of second-line treatment in order to be considered as second-line failure [21, 22]. Due to limited resources and NPH procedures VL was not tested a second time.

### Sample size

Using Stata Version 8 (Stata Cooperation, College Station, TX), a required sample size of 250 people was calculated using a 1:3 ratio between second-line and first-line children. Based on previous reports of effectiveness of first-line ART and an estimate of current VF on second-line (unpublished data), VF was expected around 6 % among second-line ART children and 25 % among first-line ART children. The sample size was adapted by estimating the number of second-line treatment patients attending the clinics over 4 months that could be enrolled (i.e.: 40–60 patients). with 10 % precision, alpha = 0.05, power 90 %.

### Data management and analysis

Data was entered in Epidata freeware (http://www.epidata.dk, Odense, Denmark) and cross-checked against original data sheets. Analyses were carried out with Stata software, Version 8 (Stata Corporation, College Station, TX, USA). Chi^2^ or Fisher’s exact test were used to assess associations between categorical variables as appropriate, and Student’s *t* test for two normally distributed continuous variables or Kruskal–Wallis as appropriate. P ≤ 0.05 was considered significant. Associations between VL and children’s characteristics were initially measured using bivariate analyses (age, sex, socio-economic conditions, schooling, time since diagnosis and ART, and adherence to treatment, initial CD4 cells count, type of treatment and weight gain over the preceding year).

Multivariate analyses between VL and children’s characteristics were conducted initially for each treatment group, then combining both treatment groups, by introducing into the model the variables significantly associated with VL (those with *p* values <0.2, Tables 1, 2). Then, a back-step selection procedure using odd ratios was used to leave only those with a *p* value <0.05 in the final model.

**Table 1:** Socio-demographic characteristics of children on first-line and second-line ART at National Pediatric Hospital

	First-line		Second-line		p
	n = 175	%	n = 57	%	
Female	91	52.0	24	42.1	0.1
Age median, years	11.4 (IQR: 9.2–13.5)		11.4 (IQR: 9.4–13.8)		0.4
Age up to 7 years	151	86.3	56	98.2	0.01
Orphan both parents	65	37.1	29	50.9	0.06
Orphan mother	10	5.7	2	3.5	0.3
Orphan father	31	17.7	8	14.0	
Living in orphanage	50	28.6	27	47.4	0.009
Child education					
Not attending school (>5 years. n = 219)	6	3.4	0	0.0	
Primary	106	60.6	43	75.4	0.2
Secondary	46	26.3	12	21.1	
Father low skilled worker (n = 191)	60	34.3	12	21.1	
Mother low skilled (n = 126)	60	47.6	15	26.3	
In charge of the child					
Mother	78	44.6	23	40.4	0.3
Father	6	3.4	1	1.8	
Grandmother	24	13.7	5	8.8	
Relatives	3	1.7	0	0.0	
Others^a^	64	36.6	28	49.1	
Family assets and characteristics					
Own their house (n = 149)	87	58.4	18	31.6	0.2
Number of people living in same house	5.1	3.3	4.6	8.1	
Daily family expense USD (n = 155)	3.3 (3.0–3.6)		3.2 (2.5–3.9)		0.4
Have a TV set	120	80.5	29	50.9	0.01
Have a car	5	3.4	1	1.8	0.8
Have a motorbike	113	75.8	27	47.4	0.8

Numbers and (percentages). Median and (interquartile range)

^a^76 were “caregivers” from the orphanage

**Table 2:** Treatment characteristics of children on first-line and second-line ART at National Pediatric Hospital

	First-line		Second-line		p
	n = 175	%	n = 57	%	
Clinical status at ART onset					
WHO Stage I	37	21.1	5	8.8	0.06
WHO Stage II	93	53.1	36	63.2	
WHO Stage III	44	25.1	14	24.6	
WHO Stage IV	1	0.6	2	3.5	
Initial CD4 cells (%)	9.1 (3.9–12.9)		7.9 (2.5–12.8)		0.1
Initial CD4 <15 %	147	84.0	52	91.2	0.17
Time before ART onset (months)	2.6 (1.1–15.7)		2.5 (1.2–8.3)		0.1
At the time of survey					
Time on ART (months)	70.5 (43.9–90.8)		81.0 (71.4–92.5)		0.001
Time since switch on second-line (months)	NA		36.5 (19.6–55.9)		
Time between VL and survey (months)	0.1 (0.1–4)		0.1(0.1–4)		0.7
Non-adherence to ART	6	3.4	0		0.1
Weight gain over 1 year (kg)	3.8 (3.5–4.2)		3.5 (2.7–4.2)		0.3
Last CD4 cells (%)	27.8 (23.6–31.8)		22 (19.1–27)		<0.001
CD4 cells <15 %	2	1.1	6	10.5	
Initial first-line treatment					
3TC + D4T + NVP	93	53.1	53	93.0	
3TC + AZT + NVP	36	20.6			
3TC + D4T + EFV	30	17.1	4	7.0	
3TC + AZT + EFV	14	8.0			
3TC + AZT + LPV	1	0.6			
3TC + D4T + LPV	1	0.6			
Current second-line treatment					
ABC + DDI + LPV			31	54.4	
3TC + TDF + LPV			13	22.8	
3TC + DDI + LPV			3	5.3	
3Tc + AZT + LPV			3	5.3	
ABC + TDF + LPV			3	5.3	
3TC + ABC + LPV			2	3.5	
3TC + AZT + LPV			2	3.5	

Numbers and (percentages). Median and (interquartile range)

*CI* Confidence interval

We have attempted to report the study according to the STROBE guidelines (Additional file 2).

## Results

### Characteristics of the study population

**Fig. 1:**
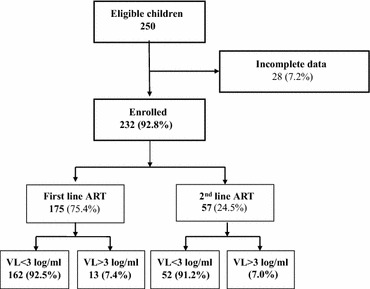
Flow chart of first and second-line ART children enrolled in National Pediatric Hospital, Cambodia

A total of 250 children (63 and 187 on second-line and first-line, respectively) were eligible for the survey. Of these, 232 (92.8 %) had VL recorded within the last 12 months and were analyzed including 175 (75.4 %) on first-line and 57 (24.6 %) on second-line ART (Fig. 1).

A total of 157 (62 %) children had a VL done during the survey including 37 (58.7 %) second-line and 120 (64.1 %) first-line. The median time between VL and survey assessment was 4.0 months (inter quartile range: 3–5).

Their social and treatment characteristics are shown in Tables 1 and 2, and supplementary tables.

Among them, 94 (40.5 %) were double orphans, 51 (22.0 %) single orphans and 77 (32.2 %) lived in orphanages. A total of 79 children (34.1 %) were cared for by their mothers, while 61 (27.6 %) by another member of the family, and 89 (38.4 %) by non-family. The median time on ART was shorter for children of the first-line group vs. for children of the second-line group (70.5 [43.9–90.8] months and 81.0 [71.4–92.5] months respectively, p = 0.001). The majority were WHO Stage II (N = 129, 55.6 %) and the median CD4 percentage in all patients was 8 % (IQR 2.9–12.9), at initiation of ART (Supplementary table). The median interval between diagnosis and ART onset was 11.8 (IQR 9.4–14.2) months. Median CD4 percentage did not differ between groups at initiation of ART (Table 2).

A total of 57 children were transferred to second-line ART within a median time of 36.5 (IQR 19.6–55.9) months. Children on second-line were more often double orphans (p = 0.06), aged over 7 years (p = 0.01) and living in orphanages than children in the first-line group. They had a significantly lower CD4 cell count at the last control.

First-line treatments began mostly with 3TC + D4T + NVP (62.9 %) while ABC + DDI + LPV (54.4 %) was the most frequent second-line regimen at the time of the survey. Full adherence to ART was self-reported for 226 children (97.4 %) and ascertained for 222 children (95.6 %) by counting pills at home or at the institution. No decrease in adherence was reported among children on second-line.

Overall, 18 (7.7 %; 95 % CI 4.6–11.9) showed a VF, 14 (8.6 %; 95 % CI 4.8–14.0) under first-line and 4 (7.0 %; 95 % CI 1.9–17.0) under second-line ART (p = 0.5).A total of 155 families (62.0 %) reported breastfeeding (61; 32.2 % vs. 31; 49.2; p = 0.04). The mean duration of breastfeeding was 7.4 months (95 % CI 7.0–7.9). Families were unable to answer the question about HIV prophylaxis during pregnancy.

The mean global weight gain within the previous year of treatment was 3.7 kg (95 % CI 3.3–4.0) without difference between the groups (3.8 kg; 95 % CI 3.4–4.1 and 3.3 kg; 95 % CI 2.0–4.5, p = 0.3, for first-line and second-line ART, respectively). The absence of weight gain over the previous year was not associated with VF. Of 6 (2.5 %) children with no weight gain, only one (0.4 %) had a VF.

In multivariate analysis, having the last CD4 percentage below 15 % was the only factor associated with VF for children on first-line treatment (OR 44.9 95 % CI 10.2–196.2) and the second-line group (OR 69; 95 % CI 4.7–995) or when combining first and second-line group together (OR 40.4; 95 % CI 11–134) (Table 3).

**Table 3:** Virological failure and associated factors for children on ART at National Pediatric Hospital (Uni and multivariate analysis)

	Success	%	Failure	%	Total	%	p	Crude			Adj.		
	N = 214	92.2	N = 18	7.8	232			OR	95 % CI	p	OR	95 % CI	p
Male	107	50.0	10	55.6	117	50.4	0.6	0.8	0.2–2.3	0.8	NI		
Female	107	50.0	8	44.4	115	49.6					NI		
Age up 7 years													
Age ≤ 7 years	24	11.2	1	5.6	25	10.8	0.4	2.1	0.3–93.4	0.7	NI		
Age > 7 years	190	88.8	17	94.4	207	89.2							
Orphan both parents													
No	135	63.1	10	55.6	145	62.5	0.5	1.3	0.4–4.0	0.6	NI		
Yes	79	36.9	8	44.4	87	37.5							
Orphan mother													
No	116	54.2	10	55.6	126	54.3	0.9	0.9	0.3–2.7	1	NI		
Yes	98	45.8	8	44.4	106	45.7							
Living status													
Living with parents	144	67.3	11	61.1	155	66.8	0.5	1.3	0.4–3.8	0.6	NI		
Living in orphanage	70	32.7	7	38.9	77	33.2							
Profession of parents													
Mother low skilled^a^													
No	48	22.4	4	22.2	52	22.4	0.9	1.0	0.2–5.3	1	NI		
Yes	69	32.2	6	33.3	75	32.3							
Father low skilled^a^													
No	23		3	16.7			0.6	0.6	0.1–4.6	0.6	NI		
Yes	66		6	33.3									
Cared for at home by													
Mother	70	32.7	9	50.0	79	34.1	*0.03*	1 (Ref.)					
Caregiver	69	32.2	7	38.9	76	32.8		0.7	0.2–2.5	0.7	NI		
Father	32	15.0	1	5.6	33	14.2		0.2	0.0–1.9	0.2			
Grandmother	30	14.0	1	5.6	31	13.4		0.5	0.0–4.1	1	NI		
Other	13	6.1	0	0.0	13	5.6		0.0	NA	0.3	NI		
House standard													
Family expense >2 USD/day													
No	19	8.9	3	16.7	22	9.5	*0.2*	0.4	0.1–2.8	0.3	NI		
Yes	195	91.1	15	83.3	210	90.5							
Have a TV set													
No	74	34.6	9	50.0	83	35.8	*0.1*	0.5	0.1–1.5	0.2	NS		
Yes	140	65.4	9	50.0	149	64.2							
Mean weight gain over last 24 months^b^	3.8 (3.4–4.1)		3.3 (2.0–4.5)				0.4				NI		
Knew his/her disease													
No	194	90.7	16	88.9	210	90.5	0.8	1.2	0.1–5.7	0.6	NI		
Yes	20	9.3	2	11.1	22	9.5							
Caregiver knows child treatment													
No	30	14.0	0	0.0	30	12.9	*0.08*	0.0	NA	0.1	NS		
Yes	184	86.0	18	100.0	202	87.1							
Initial WHO stage													
0	41	19.2	1	5.6	42	18.1	*0.1*	4.0	0.5–172	0.2	NS		
sup à 1	173	80.8	17	94.4	190	81.9							
CD4 cells <15 % (initial)													
No	31	14.5	2	11.1	33	14.2	0.6	1.3	0.2–12.7	1	NI		
Yes	183	85.5	16	88.9	199	85.8							
CD4 cells ≤15 % (last)													
No	207	96.7	7	38.8	214	92.2	<0.001	46.4	13.8–155	<0.001	40.4	11–134	<0.001
Yes	7	3.2	11	61.1	18	7.7							
Treatment													
First-line ART	162	75.7	14	72.2	175	75.4	0.7	1.1	0.3–3.7	0.4			
Second-line ART	52	24.3	4	27.8	57	24.6					NI		
Adherence	208	97.2	18	100.0	226	97.4	0.4	0.0	0–7.7	1			
Non-adherence	6	2.8	0	0.0	6	2.6							
Initial regimen with NVP													
No	48	22.4	2	11.1	50	21.6	0.3	2.3	0.5–21.3	0.3	NI		
Yes	166	77.6	16	88.9	182	78.4							
Initial regimen with D4T													
No	48	22.4	3	16.7	51	22.0	0.5	1.4	0.3–8.0	0.7	NI		
Yes	166	77.6	15	83.3	181	78.0							

Variables in italics with p < 0.2 were included in the multivariate analyses

Numbers and (percentages). Median and (interquartile range). Mean and [95 % confidence interval]

*NI* not included, *NS* non significant, *CI* Confidence interval

^a^Low skilled workers were: farmers, workers or factory workers, motor-taxi drivers, domestic helpers

^b^For children on first-line ART (OR 44.9 95 % CI 10.2–196.2) and the second-line group (OR 69; 95 % CI 4.7–995)

## Discussion

Early studies on ART in Cambodia demonstrated high effectiveness both in adults and children with follow-up ranging from 12 months to 4 years [13–15, 23–28]. At that time, reports about children’s ART chiefly expressed concerns about the challenges of detecting treatment failures using only immunological and clinical criteria [16]. Later, with the development of HIV genotypic resistance testing, high resistance mutations were reported in children with virological failure on first-line ART [17, 18]. However this testing was not yet routinely available. Our study confirms the global effectiveness of both first-line and second-line regimens for children over a long duration of follow-up (73 months), and a high level of adherence evidenced by routine tools. Second-line children who previously failed the first ART line had a similar rate of VF as children on first-line treatment. Despite these good results, a crucial issue for children with VF on second-line treatment is the lack of further options for ART in Cambodia and other countries. Besides, children on the second-line regimen had a lower median CD4 percentage at the last check. Improved measurements of adherence and access to genotypic testing for drug resistance, are urgently needed to provide high risk children with the best treatment options.

Our study confirms a high level of adherence among children currently on ART using the routine tools. Children on second-line, previously described as poorly adherent, now appear to be fully compliant with treatment. Such improvement was probably due to the implementation of home visits, called home-based care (but this issue was not documented in the survey), and the careful attention that was paid to adherence during all routine visits at the hospital. The second reason for improved adherence was probably that half of children live in orphanages which are commited to supporting adherence to ART and to routine medical follow-up. However, these good adherence results could be questioned given the occurrence of VF among children on second-line ART. The quality of adherence was probably overestimated using the routine tools (self reporting and pill counting during home visits). Health staff and the investigation team reported that parents/caregivers, being informed in advance about visits, prepared and presented to the health worker the expected number of pills. The precise reasons were not documented but could have been the fear of reprimands or that treatment would be discontinued or changed. Similar observations were reported in Ethiopia where routine adherence rates in the preceding 7 days decreased from 93.3 to 34.8 % using unannounced home-based pill counts [29]. Due to ethical concerns and fear of stigmatization [20], conducting unannounced visits was considered inappropriate by our study team.

With the 18 children who experienced a VF, it was not possible to quantify the rate of resistance to antiretroviral drugs since genotyping tests were not available, a limitation of our study. However, other studies provided information regarding HIV mutations among children with VF during the same period. Between December 2004 and January 2011, in Cambodia, all genotypic tests for HIV drug resistance mandated by the provincial capital were conducted at the Pasteur Institute. Assuming a lower threshold of VF (viral load ≥250 copies/mL), genotypic testing performed on 233 children revealed that 33 (14.1 %) harboured the Q151M and 17 (7.2 %) the K65R mutations which confer resistance to a large range of nucleoside reverse transcriptase inhibitors (NRTIs) [17]. This trend was confirmed by a similar study conducted at the Angkor Hospital for Children in Siem Reap [18]. Of 51 viremic children (>1000 copies/mL) all but one harbored drug resistance mutations to NRTIs and non-nucleoside reverse transcriptase inhibitors (NNRTIs), and half had more than 4 mutations. A quarter had multi-resistant mutations and 9 (18 %) high-level resistance-predicting mutations to subsequent therapy options including didanosine (DDI), abacavir (ABC), etravirine (ETR), and tenofovir (TDF) [18]. Emergence of these mutations is a critical issue in resource-limited settings where NRTI molecules available for second-line regimens remain limited.

The continuation of failing ART regimens is of concern since it will result in an accumulation of resistance that will hamper the effectiveness of subsequent regimens. The scaling up of drug resistance testing has been proposed [18, 28, 30].

Among the children who attended the HIV clinic, the percentage living in orphanages increased from 10 % in 2007 up to 32.2 % in 2013 [20]. In our study, the number of orphans tended to be higher among children on second-line regimens. Being an orphan was previously associated with more frequent VF [31] and subsequent transition to second-line ART. In our study, being cared for by one’s mother was a non-protective factor for VF in univariate analysis. This was probably related to the difficult situation of the families living with HIV, the majority of whom are living in extreme poverty (Table 1). So, the best way to improve ART success would probably be to improve the economic and social conditions of those living with HIV. This is supported by the protective factor (having a TV set) identified in univariate analysis, suggesting better access to information, prevention and care.

Effective interventions to reduce poverty are critical in mitigating the negative impacts of HIV and AIDS on children and households [32].

## Limitations

This study has several limitations. The first limitation is the small sample size due to time and budget constraints. The second limitation was the fact that the number and frequency of VL done per child, that could have affected the results, could not be addressed. However health staff were following the national guidelines on VL, which probably may have reduce this limitation. The third limitation was inability to conduct drug resistance tests as they were not covered by the ART program. Improving adherence support and availability of drug resistance testing are crucial to halt the rise in resistance mutations to ART drugs available in Cambodia.

## Conclusions

Children on second-line are more often double orphans, aged over 7 years and living in orphanages than children in the first-line ART in the National Pediatric hospital of Cambodia. The pattern of risk factors for VF is changing in Cambodia. The prevention of VF requires improving ART adherence and monitoring, better monitoring of children with low CD4 counts and access to routine viral load testing. Attention is needed to conflicting results of adherence and viral failure. The presence of VF in children under second-line ART is a crucial issue in a country where no third line ART is available.

## Additional files

10.1186/s13104-016-1884-y Questionnaire used during the survey.
10.1186/s13104-016-1884-y STROBE check list.

## Abbreviations

ART: antiretroviral therapy
CI: confidence interval
HIV: human immunodeficiency virus
HIV DR: HIV drug resistance
IFMT: Institut de la Francophonie pour la Médecine Tropicale
IQR: inter quartile range
OR: odds ratio
NPH: National Pediatric Hospital
NCHADS: National Center for HIV/AIDS, Dermatology and STD, Sexual Transmissible Disease
NI: not included
NS: non significant
VF: virological failure
VL: plasma HIV-1 viral Load
DDI: didanosine
ABC: abacavir
ETR: etravirine
TDF: tenofovir
