# Supplementary material for: Factors associated with the failure of first and second-line antiretroviral therapies therapy, a case control study in Cambodian HIV-1 infected children
Source: BMC Res Notes. 2016 Feb 5;9:69. doi: 10.1186/s13104-016-1884-y (PMC4744409; doi:10.1186/s13104-016-1884-y)
Supplement: Supplementary file 1 — 10.1186/s13104-016-1884-y Questionnaire used during the survey. [file 13104_2016_1884_MOESM1_ESM.docx]

**List of questions Questionnaire (translation of French questionnaire). Final questionnaire done in Khmer**

1. Code survey
2. Case-Controls 0=Controls, 1=Case
3. Agree with home visit:
4. Who answers the part of family questionnaire?

**Hospital Data (retrieved on child file)**

1. Children Hospital code
2. **Clinical status of children infected with HIV (0 = Record, 1 = Family)**
3. Date of HIV diagnosis
4. Diagnosis established in institution
5. Clinical stage at admission (WHO) (1 = stage 1, stage 2 = 2 stage 3 = 3 = 4 Stage 4)
6. Antropometry follow up

Date Date Date Date Date Date Date Date Date Date
Weight (gr)
Size (cm)

1. Initial CD4
2. **Growth follow up**

| Anthropometry | Date | Date | Date | Date | Date | Date | Date | Date | Date | Date |
| --- | --- | --- | --- | --- | --- | --- | --- | --- | --- | --- |
| Weight (gr) |  |  |  |  |  |  |  |  |  |  |
| Height (cm) |  |  |  |  |  |  |  |  |  |  |

1. Evolution of biological data

|  | Date | Date | Date | Date | Date | Date | Date | Date | Date | Date |
| --- | --- | --- | --- | --- | --- | --- | --- | --- | --- | --- |
| CD4 |  |  |  |  |  |  |  |  |  |  |
| (VL) |  |  |  |  |  |  |  |  |  |  |

**VL viral load**

**Treatment History**

1. Date first early treatment
2. Description of the treatment
3. Any change of treatment? Treatment second?
4. Date treatment.2
5. Description treatment.2
6. Reason for first change
7. Any change of treatment? Third Treatment
8. Date treatment3
9. Description treatment3
10. Any change of treatment? Reasons for changes

**Interview with child and family or gardian**

1. Date of interview
2. Child Age (year and month);
3. Date of birth if available
4. Gender: (F = 0, 1 = M)
5. Child education: (0 = No, 1 = Kindergarten, Primary = 2, 3 = Secondary)

**Marital status and family circumstances**

1. Who answers this part of questionnaire? (= 1 child, 2 = Mother, 3 = Father, 4 = Guardian, 5 = Grandmother, 9 = Others)
2. Which City area do you come from?
3. Child Status: where the child live? with the family, some one else or an institution?
4. If living with relatives,
   1. Occupation of relatives: mother / father (1 = No work, 2 = Maid, 3 = Cultivator, 4 = Worker, 5 = motorcycle taxi, 6 cop = 7 military = 8 = Merchant, 9 = Others)
   2. Education of the person giving daily care (Illiterate = 1, 2 = Primary, 3 = Secondary, 4 = Superior)
5. Who makes daily care (food, medicines) of the child? (1 = father, mother = 2, 3 = grandmother 4 = Sister 9. Other )
6. Mode of transport to get to the point of care?
7. Cost of travel to health Centre?
8. Who is the person usually accompanying the child?

**For parents or guardian only**

1. Who usually cares for the child and give the medicine?
2. Do you experience difficulties with the treatment of the child?
3. If yes, please explain and detail
4. Is one or two deceased relatives: AIDS-related deaths

**Compliance with appointment (RDV) ((For the child or with parents support)**

1. **Who answers?**
2. How many appointment in the last 6 months Missed appointment ?
3. If missed appointment for the last six months Why RDV missed?
4. If not, are there RV with unfulfilled dates?
5. If yes how many times over six months

**Related conditions and events (For the child or with parents support)**

1. Do you have any drug intolerance? Can you describe Which ones?
2. Since when?
3. Currently: does the child complain of taste and medication 0 = No, 1 = Yes?
4. **Description of ART, intake and posology**

| Name | Number | Frequency | Hour |
| --- | --- | --- | --- |
|  |  |  |  |
|  |  |  |  |
|  |  |  |  |
|  |  |  |  |
|  |  |  |  |
|  |  |  |  |
|  |  |  |  |

1. Any Forgotten or missed taking (any reason) in the last 3 days
2. If so, how often
3. Any Forgetting in the last month
4. If so, how often?
5. Overall, does the child refuse to take or spit drugs?
6. Please explain

**Scale adhesion Morisky**

1. Do you sometimes forget to take your medicine?
   People do not take their medicines regularly for other reasons that forgetting.
2. Over the past two weeks, you have not taken your medicine?
   Have you reduced or stopped taking your medication without talking to your doctor because you feel worse when you took it?
3. When travelling or leaving home, you sometimes forget to take your medication?
   Did you take all your medicines yesterday?
   When you feel that your symptoms were under control, do you
   sometimes stop taking your medication?
   Taking medicine every day is a real problem for some people. Do you feel harassed to stick to your treatment plan?
   Do you have trouble remembering to take all your medications?

**Prior Compliance (Before the last change of treatment)**

1. Forgotten or missed taking (any reason):
   If so, how often
2. Did the child refuse to take or spit drugs?

**Scale adhesion Morisky**

1. Did you sometimes forget to take your medication?
   Had you reduced or stopped taking your medication without talking to your doctor because you feel worse when you took it?
   When travelling or leaving home, you sometimes forgot to take your medication?
   When you feel that your symptoms were under control, did you sometimes stop taking your medication?
   Taking medicine every day is a real problem for some people. Did you feel harassed to stick to your treatment plan?
   Did you have trouble remembering to take all your medications?

**Knowledge of the child on his illness and its treatment**

1. Do you know your illness?
2. What is the name the disease?

Right answer (yes/no)

1. Do you know the duration of treatment?
2. If yes, for how long (duration of treatment)?
3. Do you may miss taking medication?
4. Should you take the medication when you feel well?
5. Do you know the date of appointment with the doctor
6. When is the next appointment?

**Questions to ask the mother or caretaker**

1. **Knowledge of the family or the person in charge of disease and child treatment**
2. Do you know the health of your child?
3. What is the name the disease?
4. Do you know the duration of treatment?
5. If yes, for how long (duration of treatment?)
6. Does the child may miss taking medication?
7. Should he need to take the medication when he feel well?
8. Do you know the date of appointment with the doctor
9. When is the next appointment?
10. What is the HIV status of the mother
11. Did you receive a prophylactic treatment during pregnancy
12. Did you receive a Prophylactic treatment with childbirth only
13. Did you breastfeed your child?
14. What is the ongoing treatment (ARV)?

**Stigma (For the child or with parents support)**

1. Your neighbours they know the child's illness?
2. Does the child plays with other children?
3. Is he well integrated into the neighbourhood?
4. Is it invited by others?
5. Is he left aside because of his illness?

**Other problems (For the child or with parents support)**

1. Do you have problems to report for regular visits to the hospital?
2. If so, which ones?
3. Do you have problems to report concerning the management of medicines?
4. If so, which ones?
5. Do you have problems to report concerning medication for the child?
6. If so, which ones?
7. Do you have problems to report on the safety of drugs by the child?
8. **Explain the objective of a home visit**
9. Will the family agree for a home visit?
10. If yes, Make an appointment for a home visit

**Thank you for your kind collaboration and patience**

**Part II. Home interview visit**

**Date home visit**

Confirmed child Orphan Status?

**Socio economic status (check items above that were not answered previously and respond to the following)**

1. How many people live under the same roof with you?
2. Are you the owner of the house?
2a If not, how much rent per month
3. Did you move recently?
4. Type of house (1 = slum, 2 = straw hut, 3 = bamboo, wood = 4, 5 = Apartment, 6 = Chamber)
5. How many rooms in the house
6. In the home, a) running water (tap) b) latrines c) Radio d ) TV e) BiCycle f) Moto g) car (0 = No, 1 = Yes )
7. What is the average daily expense to family eat? Check with yesterday expense: if average difficult

**Context of medical treatment**

1. Can you tell us regarding taking your treament in the last 3 days? Did you forget or miss any treatment opportunities
2. If so, how many times?
3. Oblivion on last month?
4. If so, how often
5. Does the child refuses to take medication or spits?
6. Other Problems that you like to discuss?
7. **Counting of drugs for each of drugs (ARV1, ARV2, ARV3)**
8. Date of receipt of drugs
9. Date of next meeting at the hospital
10. Name ARV1
11. Number of Days ARV1 received
12. Current number of ARV1
13. Theorical number of AV1
14. Number of ARV1 different from theorical
15. Adherence to ARV1 (%)

**Repeat for each ARV**

**Thank you for your kind collaboration and patience**
